# Supplementary material for: HIVprotI: an integrated web based platform for prediction and design of HIV proteins inhibitors
Source: J Cheminform. 2018 Mar 9;10:12. doi: 10.1186/s13321-018-0266-y (PMC5845081; doi:10.1186/s13321-018-0266-y)
Supplement: Supplementary file 2 — Additional file 2. Source code of HIVProtI web server. [file 13321_2018_266_MOESM2_ESM.zip › HIVprotI_Source-code/hivprotI/weka-3-6-6/doc/weka/filters/supervised/instance/StratifiedRemoveFolds.html]

StratifiedRemoveFolds


---


|  |  |  |
| --- | --- | --- |
| **Overview** \| **Package** \| **Class** \| **Tree** \| **Deprecated** \| **Index** \| **Help** \| **Weka's home** | |  |
| **PREV CLASS**   NEXT CLASS | **FRAMES**    **NO FRAMES**     **All Classes** |  |
| SUMMARY: NESTED \| FIELD \| CONSTR \| METHOD | DETAIL: FIELD \| CONSTR \| METHOD |  |


---


## weka.filters.supervised.instance Class StratifiedRemoveFolds

```
java.lang.Object
  weka.filters.Filter
      weka.filters.supervised.instance.StratifiedRemoveFolds
```

**All Implemented Interfaces:**: java.io.Serializable, CapabilitiesHandler, OptionHandler, RevisionHandler, SupervisedFilter

---

``` public class StratifiedRemoveFolds extends Filter implements SupervisedFilter, OptionHandler ```

This filter takes a dataset and outputs a specified fold for cross validation. If you do not want the folds to be stratified use the unsupervised version.


Valid options are:

```
 -V
  Specifies if inverse of selection is to be output.
```

```
 -N <number of folds>
  Specifies number of folds dataset is split into. 
  (default 10)
```

```
 -F <fold>
  Specifies which fold is selected. (default 1)
```

```
 -S <seed>
  Specifies random number seed. (default 0, no randomizing)
```

**Version:**
:   $Revision: 5542 $

**Author:**
:   Eibe Frank (eibe@cs.waikato.ac.nz)

**See Also:**: Serialized Form

---

**Constructor Summary**

`StratifiedRemoveFolds()`


| **Method Summary** | |
| --- | --- |
| `boolean` | `batchFinished()`             Signify that this batch of input to the filter is finished. |
| `java.lang.String` | `foldTipText()`             Returns the tip text for this property |
| `Capabilities` | `getCapabilities()`             Returns the Capabilities of this filter. |
| `int` | `getFold()`             Gets the fold which is selected. |
| `boolean` | `getInvertSelection()`             Gets if selection is to be inverted. |
| `int` | `getNumFolds()`             Gets the number of folds in which dataset is to be split into. |
| `java.lang.String[]` | `getOptions()`             Gets the current settings of the filter. |
| `java.lang.String` | `getRevision()`             Returns the revision string. |
| `long` | `getSeed()`             Gets the random number seed used for shuffling the dataset. |
| `java.lang.String` | `globalInfo()`             Returns a string describing this filter |
| `boolean` | `input(Instance instance)`             Input an instance for filtering. |
| `java.lang.String` | `invertSelectionTipText()`             Returns the tip text for this property |
| `java.util.Enumeration` | `listOptions()`             Gets an enumeration describing the available options.. |
| `static void` | `main(java.lang.String[] argv)`             Main method for testing this class. |
| `java.lang.String` | `numFoldsTipText()`             Returns the tip text for this property |
| `java.lang.String` | `seedTipText()`             Returns the tip text for this property |
| `void` | `setFold(int fold)`             Selects a fold. |
| `boolean` | `setInputFormat(Instances instanceInfo)`             Sets the format of the input instances. |
| `void` | `setInvertSelection(boolean inverse)`             Sets if selection is to be inverted. |
| `void` | `setNumFolds(int numFolds)`             Sets the number of folds the dataset is split into. |
| `void` | `setOptions(java.lang.String[] options)`             Parses a given list of options. |
| `void` | `setSeed(long seed)`             Sets the random number seed for shuffling the dataset. |

**Methods inherited from class weka.filters.Filter**

`batchFilterFile, filterFile, getCapabilities, getOutputFormat, isFirstBatchDone, isNewBatch, isOutputFormatDefined, makeCopies, makeCopy, numPendingOutput, output, outputPeek, toString, useFilter, wekaStaticWrapper`

**Methods inherited from class java.lang.Object**

`equals, getClass, hashCode, notify, notifyAll, wait, wait, wait`

**Constructor Detail**

### StratifiedRemoveFolds

```
public StratifiedRemoveFolds()
```


**Method Detail**

### listOptions

```
public java.util.Enumeration listOptions()
```

:   Gets an enumeration describing the available options..

    :   **Specified by:**: `listOptions` in interface `OptionHandler`
    :   **Returns:**: an enumeration of all the available options.

---


### setOptions

```
public void setOptions(java.lang.String[] options)
                throws java.lang.Exception
```

:   Parses a given list of options.


    Valid options are:

    ```
     -V
      Specifies if inverse of selection is to be output.
    ```

    ```
     -N <number of folds>
      Specifies number of folds dataset is split into. 
      (default 10)
    ```

    ```
     -F <fold>
      Specifies which fold is selected. (default 1)
    ```

    ```
     -S <seed>
      Specifies random number seed. (default 0, no randomizing)
    ```

    :   **Specified by:**: `setOptions` in interface `OptionHandler`
    :   **Parameters:**: `options` - the list of options as an array of strings **Throws:**: `java.lang.Exception` - if an option is not supported

---


### getOptions

```
public java.lang.String[] getOptions()
```

:   Gets the current settings of the filter.

    :   **Specified by:**: `getOptions` in interface `OptionHandler`
    :   **Returns:**: an array of strings suitable for passing to setOptions

---


### globalInfo

```
public java.lang.String globalInfo()
```

:   Returns a string describing this filter

    :   **Returns:**: a description of the filter suitable for displaying in the explorer/experimenter gui

---


### invertSelectionTipText

```
public java.lang.String invertSelectionTipText()
```

:   Returns the tip text for this property

    :   **Returns:**: tip text for this property suitable for displaying in the explorer/experimenter gui

---


### getInvertSelection

```
public boolean getInvertSelection()
```

:   Gets if selection is to be inverted.

    :   **Returns:**: true if the selection is to be inverted

---


### setInvertSelection

```
public void setInvertSelection(boolean inverse)
```

:   Sets if selection is to be inverted.

    :   **Parameters:**: `inverse` - true if inversion is to be performed

---


### numFoldsTipText

```
public java.lang.String numFoldsTipText()
```

:   Returns the tip text for this property

    :   **Returns:**: tip text for this property suitable for displaying in the explorer/experimenter gui

---


### getNumFolds

```
public int getNumFolds()
```

:   Gets the number of folds in which dataset is to be split into.

    :   **Returns:**: the number of folds the dataset is to be split into.

---


### setNumFolds

```
public void setNumFolds(int numFolds)
```

:   Sets the number of folds the dataset is split into. If the number
    of folds is zero, it won't split it into folds.

    :   **Parameters:**: `numFolds` - number of folds dataset is to be split into **Throws:**: `java.lang.IllegalArgumentException` - if number of folds is negative

---


### foldTipText

```
public java.lang.String foldTipText()
```

:   Returns the tip text for this property

    :   **Returns:**: tip text for this property suitable for displaying in the explorer/experimenter gui

---


### getFold

```
public int getFold()
```

:   Gets the fold which is selected.

    :   **Returns:**: the fold which is selected

---


### setFold

```
public void setFold(int fold)
```

:   Selects a fold.

    :   **Parameters:**: `fold` - the fold to be selected. **Throws:**: `java.lang.IllegalArgumentException` - if fold's index is smaller than 1

---


### seedTipText

```
public java.lang.String seedTipText()
```

:   Returns the tip text for this property

    :   **Returns:**: tip text for this property suitable for displaying in the explorer/experimenter gui

---


### getSeed

```
public long getSeed()
```

:   Gets the random number seed used for shuffling the dataset.

    :   **Returns:**: the random number seed

---


### setSeed

```
public void setSeed(long seed)
```

:   Sets the random number seed for shuffling the dataset. If seed
    is negative, shuffling won't be performed.

    :   **Parameters:**: `seed` - the random number seed

---


### getCapabilities

```
public Capabilities getCapabilities()
```

:   Returns the Capabilities of this filter.

    :   **Specified by:**: `getCapabilities` in interface `CapabilitiesHandler` **Overrides:**: `getCapabilities` in class `Filter`
    :   **Returns:**: the capabilities of this object **See Also:**: `Capabilities`

---


### setInputFormat

```
public boolean setInputFormat(Instances instanceInfo)
                       throws java.lang.Exception
```

:   Sets the format of the input instances.

    :   **Overrides:**: `setInputFormat` in class `Filter`
    :   **Parameters:**: `instanceInfo` - an Instances object containing the input instance structure (any instances contained in the object are ignored - only the structure is required). **Returns:**: true because outputFormat can be collected immediately **Throws:**: `java.lang.Exception` - if the input format can't be set successfully

---


### input

```
public boolean input(Instance instance)
```

:   Input an instance for filtering. Filter requires all
    training instances be read before producing output.

    :   **Overrides:**: `input` in class `Filter`
    :   **Parameters:**: `instance` - the input instance **Returns:**: true if the filtered instance may now be collected with output(). **Throws:**: `java.lang.IllegalStateException` - if no input structure has been defined

---


### batchFinished

```
public boolean batchFinished()
```

:   Signify that this batch of input to the filter is
    finished. Output() may now be called to retrieve the filtered
    instances.

    :   **Overrides:**: `batchFinished` in class `Filter`
    :   **Returns:**: true if there are instances pending output **Throws:**: `java.lang.IllegalStateException` - if no input structure has been defined

---


### getRevision

```
public java.lang.String getRevision()
```

:   Returns the revision string.

    :   **Specified by:**: `getRevision` in interface `RevisionHandler` **Overrides:**: `getRevision` in class `Filter`
    :   **Returns:**: the revision

---


### main

```
public static void main(java.lang.String[] argv)
```

:   Main method for testing this class.

    :   **Parameters:**: `argv` - should contain arguments to the filter: use -h for help


---


|  |  |  |
| --- | --- | --- |
| **Overview** \| **Package** \| **Class** \| **Tree** \| **Deprecated** \| **Index** \| **Help** \| **Weka's home** | |  |
| **PREV CLASS**   NEXT CLASS | **FRAMES**    **NO FRAMES**     **All Classes** |  |
| SUMMARY: NESTED \| FIELD \| CONSTR \| METHOD | DETAIL: FIELD \| CONSTR \| METHOD |  |


---
